# A SURVEY OF THE RADIOFREQUENCY ELECTROMAGNETIC ENERGY ENVIRONMENT IN MELBOURNE, AUSTRALIA

**DOI:** 10.1093/rpd/ncad056

**Published:** 2023-03-02

**Authors:** Stuart Henderson, Chhavi Bhatt, Sarah Loughran

**Affiliations:** Australian Radiation Protection and Nuclear Safety Agency, 619 Lower Plenty Road, Yallambie, VIC 3085, Australia; Australian Radiation Protection and Nuclear Safety Agency, 619 Lower Plenty Road, Yallambie, VIC 3085, Australia; Australian Radiation Protection and Nuclear Safety Agency, 619 Lower Plenty Road, Yallambie, VIC 3085, Australia

## Abstract

A wide variety of modern technologies make use of radiofrequency (RF) electromagnetic energy (EME) to provide convenient functions and services to users. The rise in the use of RF EME-enabled devices has led to public perception of increasing exposures and concerns about potential health effects. During March and April 2022, the Australian Radiation Protection and Nuclear Safety Agency conducted an intensive campaign to measure and characterise ambient RF EME levels within the Melbourne metropolitan area. Fifty locations across the city were visited, and a wide variety of signals in the frequency range 100 kHz to 6 GHz were detected and recorded including broadcast radio and television (TV), Wi-Fi and mobile telecommunications services. The highest measured total RF EME level was 2.85 mW/m^2^, which is equivalent to 0.14% of the relevant limit specified by the Australian Standard (RPS S-1). The results showed that broadcast radio signals were the largest contributor to measured RF EME levels at 30 locations across the suburbs, whereas downlink signals from mobile phone towers were the main contributor at the other 20 sites. Broadcast TV and Wi-Fi were the only other sources found to contribute more than 1% of the total RF EME exposure recorded at any site. All measured RF EME levels were well below the permitted limit for general public exposure given by RPS S-1 and therefore do not present a health hazard.

## Introduction

Since the introduction of amplitude modulation (AM) radio broadcasting in the early part of the twentieth century, the general Australian population has been exposed to low levels of anthropogenic radiofrequency (RF) electromagnetic energy (EME). In more recent years there has been a proliferation of consumer devices using RF EME technologies. There is a common perception in the community that RF EME exposures are increasing and may present a potential health hazard. Recognising that some members of the public are concerned, the Australian Government has supported Australian Radiation Protection and Nuclear Safety Agency (ARPANSA) to lead a research programme to conduct targeted research into RF EME issues of relevance to Australia, including measurement studies assessing RF EME levels in the community.

Surveys of particular RF EME sources have been regularly conducted by government and regulatory agencies worldwide, often at the time when new technologies are introduced^(1–5)^. The fifth generation of mobile phone technology (5G) first became operational in Australia in May 2019 and is one of the main reasons for initiation of the current measurement programme. There have been several recent RF EME surveys prompted by the global deployment of 5G; some have investigated exposures because of 5G services alone^(6, 7)^, whereas others have compared exposures because of the different mobile phone generations^(8)^.

**Figure 1:**
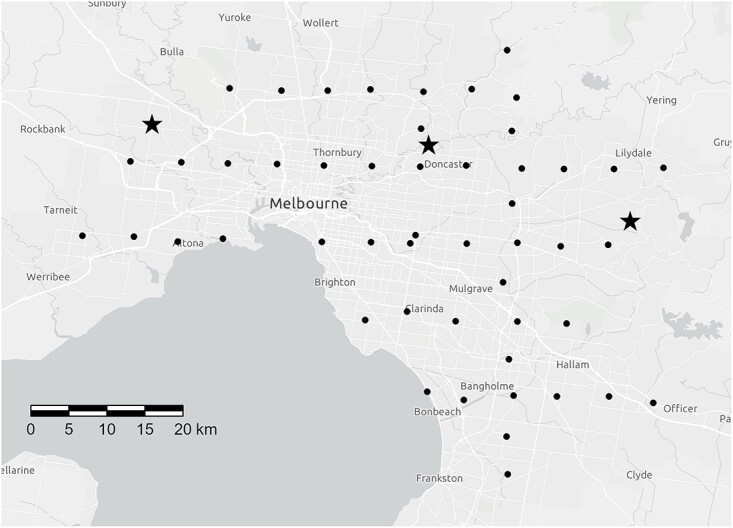
Map of Melbourne and suburbs showing locations of the 50 measurement sites (circles •) and three main broadcast transmitter sites (stars ★).

Melbourne is well-served by RF EME communications technologies. The majority of television (TV) and frequency modulation (FM) radio stations are broadcast from transmitters in the eastern outskirts of the city. There are two main AM radio transmitter sites (west and northeast of the city centre) as well as numerous other lower powered transmitter sites scattered around the metropolitan area. Locations of the main broadcast sites are shown in Figure 1. In recent years, domestic electricity metres in Melbourne have been replaced by several types of advanced metring infrastructure (AMI), often referred to as smart metres. Wireless networks (WLAN) using Wi-Fi are common in many households, business sites, schools and public areas across the city. There is also an extensive mobile phone network, with three main operators providing coverage throughout the city and its suburbs. Third generation, fourth generation and 5G (3G, 4G) mobile services are currently operating in Australia.

The purpose of this study was to conduct a series of spot measurements across the Melbourne suburban area to examine the variation in RF EME emissions and characterise the RF EME environment experienced by the general public.

## Method

A grid of 5 km squares was constructed over the Melbourne metropolitan area. For convenience, the starting location was the ARPANSA premises in Yallambie. Using this method, a total of 103 grid points were placed across the city. Google Maps was then used to identify an accessible open area suitable for RF EME measurements nearest to each grid point. Fifty of these sites were then chosen for measurement. The two longest transects, running North–South and East–West, were chosen and then four more East–West transects were selected to make up 50 measurement sites. Sites near the remaining 53 grid points will be visited as part of a future study. All measurement locations were in publicly accessible open areas. Figure 1 shows the locations of the measurement sites. All measurements were conducted on weekdays from 3 March to 27 April 2022 between the hours of 09:30 and 17:30.

At each site, a single spot measurement of actual time-averaged field levels was obtained at 1.5 m above ground in a relatively open area. The measurement protocol included broadcast services such as AM, FM and digital (DAB) radio as well as digital TV services in the very high frequency (VHF) and ultra-high frequency (UHF) bands. Also measured in this survey were non-broadcast services including VHF and UHF paging bands, industrial scientific and medical (ISM) bands used by AMI smart metres and mobile phone base station downlink bands. Bands used exclusively for mobile phone uplinks were not measured in this study. The RF EME signals were recorded over a period of 1 min in each of the frequency ranges of interest as shown in Table 1. The entire range of each of the probes was also recorded to ensure that there were no other significant contributions from services operating outside the expected bands. The maximum, minimum and mean traces were stored for subsequent analysis; in this paper, we report analysis of the mean values only. Depending on the width of the frequency range being measured, between 250 (for 5 GHz Wi Fi) and 600 (for ISM AMI) individual sweeps contributed to the stored traces.

**Table 1:** List of measured frequency bands.

Band	Frequency range [MHz]	RPS S-1 limit^a^ [W/m^2^]	Noise^b^	Sweeps^c^ per minute	Service	Technology
**H-field probe**	0.1–250		30.8 μW/m^2^	290		
AM radio	0.5265–1.6065	707	209 nW/m^2^	570	Radio	AM
**E-field probe**	75–3000		27.7 μW/m^2^	90		
FM radio	87.5–108	2.00	903 nW/m^2^	520	Radio	FM
VHF	148–174	2.00	561 nW/m^2^	510	Paging	
VHF Band III	174–230	2.00	844 nW/m^2^	600	TV and radio	DVB-T DAB
UHF	403–420	2.02	122 nW/m^2^	530	Paging	
**E-field probe**	420–6000		80.6 μW/m^2^	50		
UHF	450–520	2.25	3.32 μW/m^2^	360	Paging	
UHF Band IV	526–582	2.63	2.14 μW/m^2^	600	TV	DVB-T
BTS 700 MHz DL	758–803	3.79	976 nW/m^2^	390	Mobile BTS	4G/LTE
BTS 800 MHz DL	870–890	4.35	305 nW/m^2^	520	Mobile BTS	3G/WCDMA 4G/LTE 5G/NR
ISM (AMI)	915–928	4.58	181 nW/m^2^	610	Smart metres	
BTS 900 MHz DL	935–960	4.68	339 nW/m^2^	510	Mobile BTS	3G/WCDMA NB-IoT
BTS 1800 MHz DL	1805–1880	9.03	227 nW/m^2^	520	Mobile BTS	4G/LTE GSM-R
DECT	1880–1900	9.40	60.8 nW/m^2^	520	Cordless phones	DECT
BTS 2100 MHz DL	2110–2170	10	61.6 nW/m^2^	430	Mobile BTS	3G/WCDMA
BTS 3.4 GHz TDD	3425–3700	10	441 nW/m^2^	390	Mobile BTS	5G/NR
WLAN 5 GHz	5150–5850	10	2.11 μW/m^2^	250	Wi-Fi	

^a^The most restrictive general public exposure limit for each frequency range is based on the reference levels for whole-body exposure as specified in the Australian standard RPS S-1^(9)^.

^b^Noise values were measured in an RF-shielded room.

^c^Typical number of sweeps obtained in the 1-min measurement period.

RF EME measurements were obtained using a handheld spectrum analyser (Narda SRM 3006) connected via a 1.5 m long ferrite beaded RF cable to either a H-field (Narda 3581/01) or one of two E-field probes (Narda 3501/01 and 3502/01) mounted at 1.5 m above ground level on a non-conductive tripod. All of the probes had tri-axial sensors. The spectrum analyser was operated using predefined measurement routines, which we set up to perform an average over 1 min for each frequency range of interest, recording the root-sum-squared of the output from the probe’s three axes. The use of measurement routines ensured the consistency of the settings at all sites independent of the person operating the instrument. Researchers conducting the measurements disabled their personal transmitting devices during the acquisition of measurements.

As the SRM-3006 is a frequency selective instrument it permits analysis of RF EME levels in specific frequency ranges. Along with the knowledge of the permitted use of spectrum in licenced bands this allows the attribution of RF EME levels to particular services and technologies. In this study, we did not perform code-selective measurements (which would enable attribution to particular mobile phone towers) as the purpose of this study was to determine typical exposures because of all measurable sources.

Measurements of RF signals are always subject to background (thermal) noise of the detector and associated metre electronics. When integrating over wide frequency ranges the low-level background noise can add up to a significant value if due care is not taken when selecting the equipment settings. Prior to obtaining RF EME measurements in the field, the equipment was operated in a (RF) shielded laboratory at ARPANSA using the same equipment settings employed for the survey measurements in the field. In this way, we were able to determine the typical background noise values for the equipment (see Table 1). The criteria we chose for determining the existence of detectable signals in a frequency band were that the measured power must exceed the noise level by at least 10% and the net power must be >1 nW/m^2^.

Results are reported as equivalent plane wave power flux densities in units of W/m^2^ for all services. The AM radio measurements were obtained using the H field probe that responds to magnetic field strengths (A/m) and are converted to units of W/m^2^ by the spectrum analyser. All other service bands were measured using one of two E-field probes that respond to electric field strengths (V/m) before being converted to units of W/m^2^ by the spectrum analyser. Comparisons are made with the reference levels for general public time-averaged whole-body exposure limits given by the ARPANSA Standard RPS S 1^(9)^. This Standard is based on the ICNIRP (2020) recommendations for RF fields^(10)^. Where the limit varies across the frequency range occupied by the service, the most restrictive limit within the frequency range is used.

It is assumed that all exposures occur in the far-field of the antennas. While this is certainly reasonable for all services above 10 MHz, it is only for the AM radio measurements that more caution is required. The AM Radio band uses the frequency range 526.5–1606.5 kHz in Australia, resulting in far-field boundaries of around 190–600 m from the antenna. Of all the measurement locations visited in this study none were closer than 2 km from the nearest AM Radio broadcast site. Since the far-field conditions are satisfied, the measured magnetic field strength, after conversion to an equivalent plane wave power flux density, can be compared with the most restrictive H^2^ equivalent power density reference level derived from the magnetic field reference level in this frequency range.

Measured values from each of the frequency bands were grouped by service, according to the arrangement shown in Table 1. For instance, AM, FM and DAB Radio services are transmitted in three different frequency bands but all provide Radio services. The mobile phone base station signals were grouped according to the technology being used: 3G/WCDMA, 4G/LTE, 5G/New Radio (NR), Narrowband Internet of Things (NB-IoT), Global System for Mobile Communications—Railway (GSM-R).

The Radio Frequency National Site Archive (RFNSA) is a publicly accessible database listing the locations and technical details of all the mobile phone base stations (base transmit station, BTS) in Australia^(11)^. Data extracted from the RFNSA was used to determine the locations of BTS relative to the 50 measurement locations.

## Results

Radio and Mobile BTS signals were detected at all 50 sites and only two sites did not have any detectable TV signals. The other services (paging, AMI, cordless phones (DECT), Wi-Fi) were less often found at measurable levels. Table 2 shows the number of sites at which each service type was detected.

**Table 2:** Number of sites at which each service type was detected.

Service type	Number of sites
Radio	50
TV	48
Paging	23
Mobile BTS	50
AMI smart metres	11
DECT cordless phones	10
Wi-Fi	28

Results showed that 88% of the measurement sites had at least one BTS within 1 km (only six did not), whereas 50% of sites had at least one BTS within 500 m. The median number of BTS within 1 km of the measurement location was 2.5 (inter quartile range 4), whereas for one site there were 44 BTS within 1 km. The median distance to the nearest BTS was 515 m (inter quartile range 350 m), with the closest being at 150 m and the furthest at 1520 m from the measurement site.

**Figure 2:**
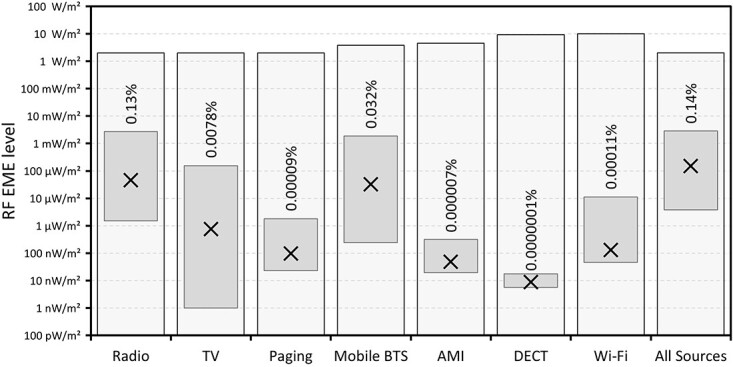
Range (floating columns) and median (crosses) of 1-min average RF levels measured at each of the 50 sites classified by service. Also shown is the general public exposure limit (shaded columns) and the highest measured value as a percentage of that limit.

**Figure 3:**
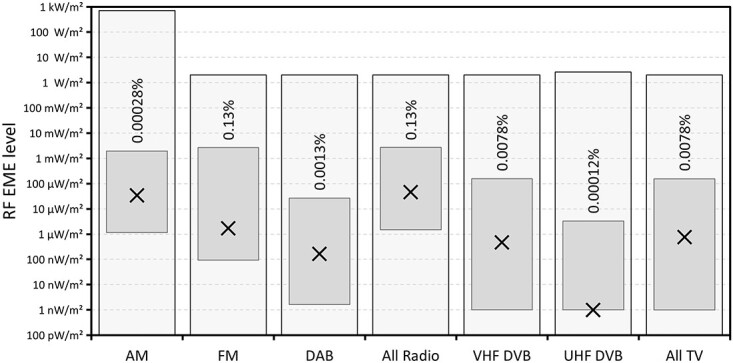
Range (floating columns) and median (crosses) of 1-min average RF levels measured at each of the 50 sites from broadcast radio and TV services. Also shown is the general public exposure limit (shaded columns) and the highest measured value as a percentage of that limit.

**Figure 4:**
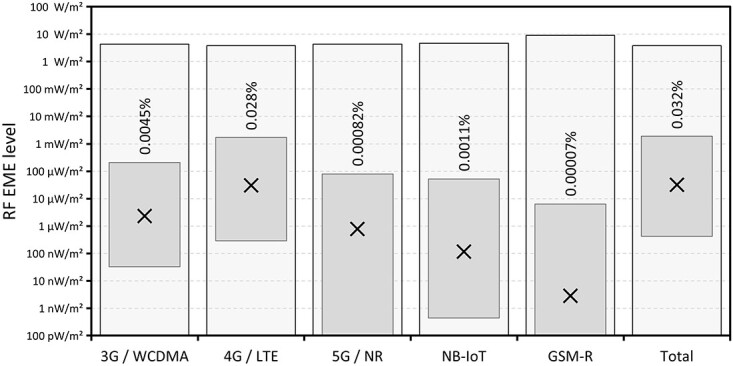
Range (floating columns) and median (crosses) of 1-min average RF levels measured at each of the 50 sites from mobile phone base station services. Also shown is the general public exposure limit (shaded columns) and the highest measured value as a percentage of that limit.

The range and median values of RF EME levels measured across the 50 sites are shown in Figures 2–4 along with the relevant general public exposure limit and the highest measured value as a percentage of that limit. Figure 2 shows the values classified by service. Broadcast radio and TV services, separated by transmission technology, are shown in Figure 3. While Figure 4 shows the values because of mobile phone base station services classified by technology.

## Discussion

The results of this study provide a direct measure of the typical RF EME exposure levels encountered by the general public.

The maximum and median Radio and Mobile BTS values measured were comparable across sites, although the Radio signals represented a higher proportion of the permitted limit value. AMI smart metres and DECT cordless phones contributed very little to the measured RF EME in this study, which was unsurprising given the measurement protocol specified outdoor locations in open areas and few measurement locations were very close to houses, offices or other buildings where these sources are located. The AMI devices used in Australia employ a mesh radio system that operates in the 915–928 MHz ISM band along with a variety of other low power devices. The AMI radio transmitter is typically of 1 W power and each device is only active periodically for a very short duration (typical duty cycle < 1%)^(12)^. Signals from AMI smart metres were seen at only 11 of the 50 locations, whereas DECT cordless phones were observed at just 10 of the 50 locations. While paging services and Wi-Fi signals were recorded at many of the locations, the RF EME attributed to these sources was also quite modest, far lower than that because of broadcast radio and BTS services. This is likely because of the intermittent nature of paging signals and the relatively short range and low power of Wi-Fi.

The median RF EME across all 50 sites from all sources was 150 μW/m^2^, equivalent to 0.0011% of the general public exposure limit. The highest measured total RF EME level (the sum of 1-min mean values from all sources at a single location) of 2.85 mW/m^2^ was found in the suburb of The Basin. This reading is equivalent to 0.14% of the allowable limit, or more than 700 times below the limit. The Basin is located at the foot of Mt Dandenong, which hosts the main broadcast TV and FM radio transmitters for the city. Unsurprisingly, RF EME from radio and TV services dominated the total exposure at this site contributing 94% and 5% of the total measured RF EME, respectively.

Maximum values from broadcast AM and FM radio services were comparable; however, the median AM radio value was more than an order of magnitude higher than the median FM radio value. This is quite different to the situation reported for the USA, where FM radio is far more dominant, and the contribution of AM radio was found to be insignificant^(13)^. Digital radio (DAB) was much lower than both AM and FM radio. The maximum and median values due to VHF TV were much higher than the contributions from UHF TV services, but still lower than the levels attributed to both AM and FM radio.

There are a number of different sub 6 GHz frequency bands currently being used from 700 MHz to 3.6 GHz to host mobile phone services. Millimetre wave 5G services are also beginning to be introduced in the 26 GHz band, although these frequencies were not measured in this study. There are often multiple technologies used in a single frequency band, so care must be taken to identify the signal type and attribute it to the correct technology. Detected signals from BTS were generally dominated by 4G services. Of the three technologies in commercial operation at the time of this study, the services were ranked 4G, 3G then 5G in decreasing level for both the maximum and median values. Significant GSM-R signals were only detected at sites close to train lines. Only one NB-IoT service was detected in Melbourne (found at 48 sites), which was operating in the 900 MHz band.

Measurements reported in this paper reflect the cumulative RF EME levels due to all significant sub 6 GHz sources at the time of measurement. No attempt has been made to extrapolate to a theoretical worst-case maximum exposure condition. This decision was taken as extrapolation to the maximum possible RF EME levels is both difficult and time-consuming, particularly when not targeting a specific BTS, while providing little further insight into realistic exposure levels. The absolute maximum output state of the mobile phone network represents an unrealistic condition that is extremely unlikely to ever be realised in practice^(14)^.

The results from this survey are broadly consistent with those reported elsewhere, although direct comparison is often difficult because of variations in measurement protocols. Amongst the other studies that included broadcast services as well as mobile phone base stations, our results for the highest mean total RF EME level of 2.85 mW/m^2^, equivalent to 0.14% of the allowable limit, are remarkably similar to those reported by Tell and Kavet^(13)^ who found 2.5 mW/m^2^ (0.12% of the allowable limit) in the USA and comparable to those of Verloock *et al*.^(15)^ who found 1.02 mW/m^2^ (% of limit not reported) in Belgium and van Wyk *et al*.^(16)^ 955 μW/m^2^ (0.016% of the allowable limit) in South Africa.

As 5G NR services are introduced, surveys are being conducted by other government and regulatory agencies both in Australia and other countries^(1–5,17)^. Reported levels vary greatly depending on the measurement protocol and the active status of the 5G network. In this study, we did not target locations expected to have high exposure levels because of nearby base stations, perform extrapolation to full traffic conditions or attempt to attract 5G NR beams. As a consequence, our measured values (<100 μW/m^2^) attributed to 5G NR are much lower than the values reported in other recent studies where the 5G BTS was intentionally 100% loaded and the measurement extrapolated (Aerts *et al*.^(18)^ 37 mW/m^2^ and Aerts *et al*.^(7)^ 49 mW/m^2^). They are, however, similar to levels reported by Deprez *et al*.^(6)^ in the absence of active user equipment (86 μW/m^2^).

Many different measurement protocols have been employed when investigating environmental levels of RF EME. There is often variation between studies in the choice of height for the measurement, the use of spatial averaging techniques and the duration of time averaging periods. Positioning the probe at a height of 1.5 m above ground level is typical when characterising human exposure^(7,15)^ and it is also commonly used as one of the heights for spatial averaging schemes^(16)^. The averaging times referred to in exposure standards (continuous 6- or 30-min periods) are based on tissue heating phenomena, not on the variation of radiocommunication signals, and are not necessarily the same as the measurement times needed to estimate field strengths^(9,10)^. Shorter averaging times, ranging from 30 s to 2 min, have been shown to be sufficient to derive field values that are accurately representative of longer averaging times^(6,7,13,18)^. We believe our choice of a 1.5 m measurement height and an averaging time of 1 min provides a realistic assessment of the RF EME environment while also being practical to implement.

Building on this current work, ARPANSA will continue to monitor RF EME levels in the Australian environment. Future work will address some of the limitations of this study. This will include conducting surveys targeting locations where exposures to the general public from mobile phone base stations are expected to be highest, rather than simply measuring at pre-defined grid points. As travel restrictions because of the pandemic have been removed, measurement surveys will be conducted in regional towns and other cities. As new measurement equipment becomes available, future surveys will also evaluate RF EME exposures from the 5G-NR services employing millimetre waves that are currently being introduced in Australia. Further analysis of the current data set will assess how the ambient RF EME levels measured in this study compare with earlier survey results collected at some of the same sites in the early 2010s.

## Conclusion

This study presented data from measurements conducted at 50 sites characterising the ambient RF EME levels within the metropolitan area of the Australian city of Melbourne. The largest recorded values were due to broadcast radio services and mobile phone network downlinks. Across all sites, these two services had comparable maximum and median values. RF EME from broadcast services was overwhelmingly due to AM radio, whereas RF EME from mobile phone base stations was predominantly due to 4G services. All measured RF EME values were far below the permitted limits for public exposure from the relevant Australian standard, and hence, do not present a risk to citizens’ health.

## Data Availability

The data underlying this article will be shared on reasonable request to the corresponding author.
